# The Effect of Radioiodine Therapy on the Prognosis of Differentiated Thyroid Cancer with Lung Metastases

**DOI:** 10.3390/biomedicines12030532

**Published:** 2024-02-27

**Authors:** Shenghong Zhang, Mengqin Zhu, Han Zhang, Hanhui Liu, Xin Fan, Jiajia Zhang, Fei Yu

**Affiliations:** 1 Shanghai Clinical College, Anhui Medical University, Shanghai 200040, China; hailee6677@163.com (S.Z.);; 2 The Fifth Clinical Medical College, Anhui Medical University, Hefei 230032, China; 3 Department of Nuclear Medicine, Shanghai Tenth People’s Hospital, Tongji University School of Medicine, Shanghai 200040, China; 4 Institute of Nuclear Medicine, Tongji University School of Medicine, Shanghai 200040, China

**Keywords:** lung metastasis, RAI, thyroid cancer, thyroid cancer-specific survival

## Abstract

Lung metastasis substantially influences the survival of thyroid cancer (TC) patients. This study sought to investigate factors impacting the survival of differentiated thyroid cancer patients with lung metastases (DTC–LM) undergoing radioiodine therapy (RAI) after thyroid surgery. The retrospective study encompassed 609 TC patients with lung metastases. Survival outcomes—specifically, overall survival (OS) and thyroid cancer-specific survival (TCSS)—were examined through both univariate and multivariate Cox regression analyses. Radioiodine therapy (RAI)’s impact on DTC–LM patient survival was further assessed with the Kaplan–Meier survival curve. Of the 609 TC patients with lung metastases, 434 (71.3%) were found to have undergone thyroid surgery after a median follow-up of 59 months. Anaplastic thyroid cancer (ATC), stage IV, and lung metastases associated with other metastases were identified as risk factors for OS and TCSS in TCLM patients. RAI therapy significantly enhances survival in DTC–LM patients followed by primary site surgery under the age of 55, PTC patients, and those with single organ metastases at lung.

## 1. Introduction

Thyroid cancer (TC) is the most prevalent malignant tumor in the head and neck region, originating from either the thyroid follicular epithelium or parafollicular epithelial cells [1,2]. According to the latest cancer report published in the Journal of the National Cancer Center (JNCC), the incidence of TC in China has increased 20-fold between 2000 and 2016 [3]. TC is histologically categorized into differentiated thyroid cancer (DTC), medullary thyroid cancer (MTC), and anaplastic thyroid cancer (ATC), based on differences in tumor origin and differentiation [4]. Papillary thyroid cancer (PTC) and follicular thyroid cancer (FTC) are two subtypes of DTC, with the former being the most common subtype, accounting for approximately 90% of all TC cases [5,6].

The overall prognosis for patients with DTC is favorable, while patients with distant metastases have a significantly poorer prognosis [7,8]. The lung is the most commonly affected site of distant metastases for DTC metastases, with an incidence rate of 2–20% [9,10]. Indeed, research indicated a higher incidence of lung metastasis compared to other organs in thyroid cancer patients with distant metastasis [11,12,13], likely due to the lungs’ extensive vascularization and tumor-friendly microenvironment [14]. Of those with lung metastases, 50% of patients die within 10 years [9,15,16]. Several factors, including age, serum thyroglobulin levels, cumulative dose of radioiodine therapy (RAI), and tumor size, have been shown to influence the prognosis of patients with DTC correlated with pulmonary metastases [17,18]. Despite the considerable number of studies on patients with lung metastases from thyroid cancer, few studies have focused on the prognostic impact of all pathological types on thyroid cancer with lung metastasis (TCLM).

The therapeutic approaches for TC primarily comprise surgical intervention, RAI therapy, external radiotherapy, and chemotherapy, with targeted therapy and immunotherapy emerging as novel systemic treatment modalities in recent years [19,20]. The 2015 guidelines of the American Thyroid Association (ATA) highly endorse RAI therapy as the recommended treatment following thyroidectomy for stratified DTC patients at high risk of recurrence [21]. Additionally, patients with ^131^Iodine-avid lung metastasis, smaller nodules, no other metastases, and younger age groups (<55 years) demonstrate favorable prognosis [22,23]. However, the factors influencing the survival of patients with TCLM who have undergone primary site surgery and RAI therapy necessitate further exploration.

In this study, our primary objective was to identify prognostic risk factors for TCLM patients who have undergone primary site surgery based on the Surveillance, Epidemiology, and End Results (SEER) database. The impact of RAI therapy on the survival of TCLM patients who have undergone primary site surgery was also evaluated through a stratified analysis based on age, histology, and other distant metastases. These findings could prove valuable in guiding clinical decision-making related to therapy.

## 2. Materials and Methods

### 2.1. Data Source

This retrospective study employed data from the SEER program, which covers around 30% of the entire U.S. population. We identified patients diagnosed with 87,737 cases of thyroid cancer between 2010 and 2017 using SEER*stat 8.3.5 software. The year 2010 was chosen as the starting point due to the availability of data on distant metastases beginning from that year. Furthermore, the American Joint Committee on Cancer (AJCC), 7th edition was utilized for the years 2010 to 2015. From 2016 to 2017, cancer registries in the United States transitioned from Collaborative Staging (CS) to SEER Combined Stage (2016–2017), which incorporates TNM categories, staging groups, and definitions based on the TNM Classification of Malignant Tumors, 7th edition. As such, all AJCCs from 2010 to 2017 are referred to as AJCC 7th. Given the variability of AJCC 8th and constraints on SEER data collection, there is currently no viable conversion method, and, hence, we opted for the unification of SEER data collection while utilizing AJCC 7th.

### 2.2. Patient Selection

**Figure 1:**
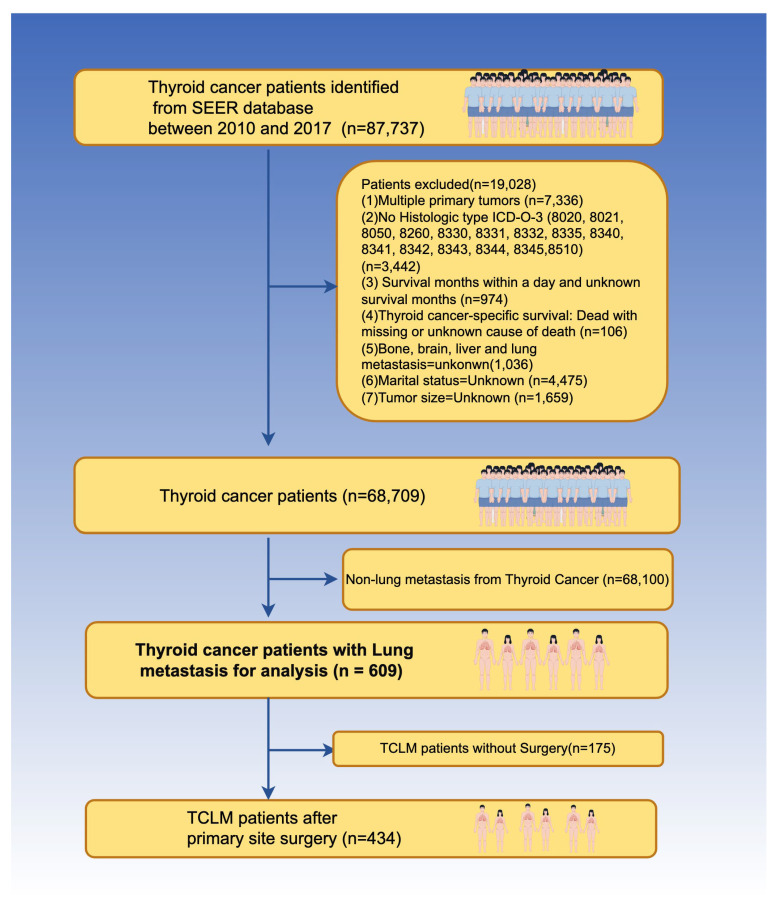
The schematic workflow of patient selection (By Figdraw). SEER: The Surveillance, Epidemiology, and End Results; TCLM: Thyroid cancer with lung metastasis.

The study retrieved data from the SEER database, which included variables such as race, sex, marital status, histological type ICD-O-3 [PTC (8050, 8260, 8340, 8341, 8342, 8343, 8344), MTC (8345, 8510), FTC (8330, 8331, 8332, 8335), ATC (8020, 8021)] [24], T stage, N stage, SEER-specific etiology of death classification, vital status, and survival time (months). Other races encompassed American Indian/Alaska Native, Asian or Pacific Islander, and unknown racial groups, due to their representation of only 1% of the SEER database. TNM staging and stage data were based on a combination of derived AJCC 7th (2010–2015) data and derived SEER combined TNM data due to constraints in the composition of the SEER database across years. Exclusions were made for TC patients with unknown demographic and clinicopathological information from 2010 to 2017. Despite marital status being a significant determinant in various cancers, its significance in thyroid cancer remains unknown. Therefore, patients with uncertain marital status were eliminated to investigate the impact of marital status on the prognosis of TC patients [25,26]. Thyroid cancer patients were classified into two groups: those with and without lung metastases. In TCLM patients, an additional distinction was made based on whether primary surgery was performed. The detailed screening process is illustrated in Figure 1.

As all data used in this study were obtained from the publicly available SEER database, no approval from a medical ethics committee was necessary since the database is accessible to the public free of charge. Therefore, the Ethics Committee of Shanghai Tenth People’s Hospital deemed that no review of this study was required.

### 2.3. Statistical Analyses

First of all, the study employed the chi-square test to compare the demographic and clinical features of TCLM patients with and without radioiodine therapy. Secondly, univariate and multivariate Cox regression analyses were conducted to identify the factors that impact overall survival (OS) and thyroid cancer-specific survival (TCSS) in patients with TCLM, as well as the possible factors that affect prognosis in TCLM patients after primary site surgery. The results were presented as hazard ratio (HR), 95% confidence intervals (CI), and *p*-value. Finally, the stratified Kaplan–Meier survival curve was utilized to investigate the parameters that influence TCSS with RAI therapy in TCLM patients who underwent primary site surgery. A *p*-value of 0.05 was considered statistically significant. All data were analyzed using SPSS software (version 26; IBM Corp., Armonk, NY, USA).

## 3. Results

### 3.1. Demographic and Clinicopathologic Characteristics of Participants 

This study involved 68,709 patients diagnosed with TC between 2010–2017. After a median follow-up of 59 months, the patients were categorized into two groups: those with pulmonary metastases and those without. The sex ratio was approximately 1:1, while the proportion of patients aged over 55 years was higher in the TC patients with lung metastasis compared to those without. More than half of the cases were classified as PTC and had a tumor size > 40 mm. Furthermore, the proportion of TC patients with lung metastasis who underwent primary site surgery was notably higher than those who did not receive surgery at the primary site, accounting for 71.3% and 28.7%, respectively. Additionally, among the TC patients with lung metastasis (LM), 36.6% underwent RAI treatment, while 63.4% did not (Supplementary Table S1). Among TCLM patients who underwent RAI treatment, 99.6% opted for surgical intervention at the primary site. Table 1 provides further details of the demographics and clinicopathologic characteristics of thyroid cancer patients with lung metastases.

### 3.2. The Predictive Factors of Overall Survival and Thyroid Cancer-Specific Survival in Patients with TCLM

In the univariate Cox analysis, several factors emerged as significant risk factors for the OS of patients with TCLM. These factors included age ≥ 55 years (HR = 2.474, 95%CI: 1.885–3.246, *p* < 0.001), non-PTC (MTC vs. FTC vs. ATC, 2.233 (1.350–3.695) vs. 1.578 (1.118–2.229) vs. 10.827 (8.256–14.200), *p* < 0.05), tumor size > 40 mm (HR = 2.868, 95%CI: 1.566–5.252, *p* < 0.05), stage IV (HR = 7.143, 95%CI: 3.915–13.036, *p* < 0.001), T4 (HR = 4.238, 95%CI: 2.425–7.405, *p* < 0.001), and LM combined with other distant metastasis (Lung + 1 vs. Lung + 2, 1.635 (1.282–2.084) vs. 2.505 (1.741–3.604), *p* < 0.001). Moreover, surgical intervention at the primary site (HR = 0.223, 95%CI: 0.179–0.278, *p* < 0.001 for OS; HR = 0.224, 95%CI: 0.178–0.282, *p* < 0.001, for TCSS) was found to have a potentially positive effect on both OS and TCSS, while sex did not. RAI therapy (HR = 0.188, 95%CI: 0.142–0.248, *p* < 0.001, for OS; HR = 0.169, 95%CI: 0.124–0.229, *p* < 0.001, for TCSS) improved overall survival (OS) and thyroid cancer-specific survival (TCSS) of TCLM patient, which can effectively manage the progression of TCLM patients.

In the multivariate Cox analysis, three variables were identified as significant risk factors for the OS of TCLM patients. These variables included ATC (HR = 3.981, 95%CI: 2.876–5.512, *p* < 0.001, for OS; HR = 3.864, 95%CI: 2.763–5.403, *p* < 0.001, for TCSS), stage IV (HR = 2.794, 95%CI: 1.433–5.447, *p* = 0.003, for OS; HR = 2.768, 95%CI: 1.379–5.556, *p* = 0.004, for TCSS), and lung metastasis combined with other metastasis (Lung + 1, 1.299 (1.002–1.684), *p* < 0.05, for OS; Lung + 1,1.359 (1.039–1.779), *p* < 0.05, for TCSS). Conversely, both surgical intervention at the primary site (HR = 0.653, 95%CI: 0.506–0.842, *p* = 0.001, for OS; HR = 0.664, 95%CI: 0.510–0.864, *p* = 0.002, for TCSS) and RAI therapy (HR = 0.397, 95%CI: 0.283–0.558, *p* < 0.001, for OS; HR = 0.371, 95%CI: 0.257–0.535, *p* < 0.001, for TCSS) were considered protective factors for the OS and TCSS of TCLM patients. Table 2 presents the HR and 95%CI for each variable in both the univariate and multivariate Cox proportional hazard models.

### 3.3. The Predictive Factors of OS and TCSS in TCLM Patients following Primary Site Surgery 

To further investigate the prognostic factors of patients with TCLM who have undergone surgery at their primary site, a multivariate Cox analysis was conducted. The results of the analysis revealed that age (HR = 1.484, 95%CI: 1.018–2.165, *p* < 0.05, for OS), histology (ATC, 4.672 (2.964–7.366), *p* < 0.001, for OS; ATC, 4.626 (2.890–7.405), *p* < 0.001, for TCSS), stage (IV, 2.631 (1.238–5.592), *p* < 0.05, for OS; IV, 2.589 (1.170–5.728), *p* < 0.05, for TCSS), T stage (T4, 3.133 (1.366–7.187), *p* < 0.01, for OS; T4, 3.358 (1.386–8.133), *p* < 0.01, for TCSS), and other distant metastasis (Lung + 1 vs. Lung + 2, 1.551 (1.097–2.194) vs. 1.866 (1.064–3.274), *p* < 0.05, for OS; Lung + 1 vs. Lung + 2, 1.725 (1.207–2.464) vs. 1.837 (1.026–3.288), *p* < 0.05, for TCSS) were all significant predictors of OS in these patients. The risk of OS in TCLM patients who received RAI treatment after primary site surgery was 0.507 times that of no treatment (HR = 0.507, 95%CI: 0.350–0.733, *p* < 0.001). Total thyroidectomy, subtotal thyroidectomy or near-total thyroidectomy at the primary site accounted for 95.0% of TCLM patients treated with RAI therapy (Supplementary Table S2). Similarly, these same variables, with the exception of age (*p* = 0.103), were also found to be independent predictors of TCSS in TCLM patients following primary site surgery. As a result, TCLM patients who were older than 55 years, had an ATC subtype, were in stage IV, had T4 stage, had multiple distant metastases, and did not receive RAI treatment were shown to have a poorer prognosis (Table 3). TCLM patients who receive RAI therapy after primary site surgery are associated with a diminished risk of TCSS relative to their untreated counterparts (HR = 0.482, 95%CI: 0.323–0.718, *p* < 0.001).

### 3.4. Effect of RAI on Survival in Subgroup

To analyze the difference in survival among TCLM patients who underwent surgery at the primary site, the Kaplan–Meier method and log-rank test were utilized. The Kaplan–Meier analysis was stratified based on RAI treatment, age, histology, and other distant metastases.

**Figure 2:**
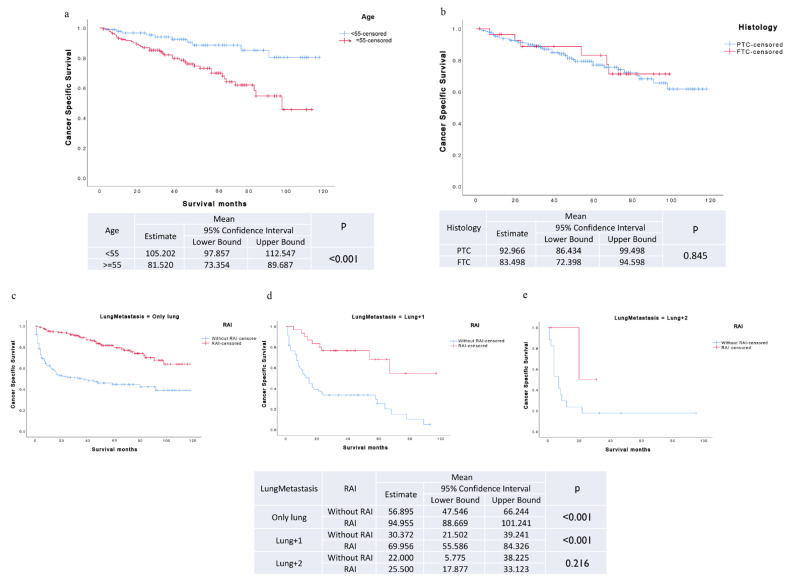
Stratified Kaplan–Meier survival curves of TCSS based on subgroups of age, histology, and lung metastasis. (**a**) In TCLM patients who underwent RAI treatment following primary surgery, younger patients (age < 55 years) exhibited enhanced TCSS compared to older patients (age ≥ 55 years). (**b**) Among TCLM patients who underwent primary site surgery and received RAI therapy, no statistical difference in TCSS was observed between PTC patients and FTC patients. (**c**–**e**) Among TCLM patients who underwent primary site surgery and received RAI therapy, those with only lung metastasis exhibited superior TCSS compared to those with a combination of lung and one other distant metastasis. Only lung: Thyroid cancer patients with single organ metastases at lung; Lung + 1: Thyroid cancer patients combined with lung and one other distant metastasis; Lung + 2: Thyroid cancer patients combined with lung and other several distant metastases.

Firstly, improved TCSS was observed in patients with TCLM who underwent RAI treatment post-primary surgery compared to those who did not receive RAI, irrespective of age. In TCLM patients who underwent RAI treatment following primary surgery, younger patients (age < 55 years) exhibited considerably enhanced TCSS in contrast to their older counterparts (age ≥ 55 years) (Figure 2a and Figure S1a,b).

Secondly, regardless of whether the histology is PTC or FTC (ATC and MTC were not considered because patients with them rarely received RAI treatment), TCLM patients who underwent surgery of the primary site followed by RAI treatment had significantly better prognosis compared to those without RAI. However, the histology did not affect the impact of RAI on the TCSS of TCLM patients following surgery at the primary site (Figure 2b and Figure S1c,d).

Last but not least, the benefits of RAI therapy on the TCSS of TCLM patients following primary site surgery were observed in subgroups with only lung metastasis (*p* < 0.001) and those with a combination of lung and one other distant metastasis (*p* < 0.001). However, this effect was not observed in TCLM patients who had multiple distant metastases following primary site surgery. Notably, among TCLM patients who underwent primary site surgery and received RAI therapy, those with only lung metastasis exhibited superior TCSS compared to those with a combination of lung and one other distant metastasis (Figure 2c–e).

## 4. Discussion

The results of this study indicate that surgery at the primary site may serve as an independent prognostic factor to improve the OS and TCSS in patients with TCLM. A previous study reported that surgery at the primary site, whether for the primary or distant site, can prolong OS and TCSS in TCLM patients [27]. Additionally, our study suggests that combining surgery of the primary site with RAI therapy may further enhance the prognosis of patients with lung metastasis compared to surgery of the primary site alone. These findings underscore the importance for clinicians to carefully consider the appropriate surgical approach and adjuvant therapy for lung metastasis, factoring in the patient’s overall condition.

RAI therapy has been identified as a highly efficacious intervention for the management of metastatic DTC, offering a substantial extension of life expectancy for patients [28]. Our results demonstrate that RAI therapy confers a significant improvement in both overall survival rate and thyroid cancer-specific survival rate for patients with TCLM compared to those who do not receive RAI therapy. Despite the considerable number of studies that examine the impact of RAI therapy on the prognosis of DTC patients with lung metastasis [11,29], few studies have reported the effect of RAI therapy on the prognosis of all histological types of thyroid cancer with lung metastasis. Therefore, this study provides a systematic analysis of the factors influencing the survival of patients with this condition. 

Firstly, this study has revealed that nearly 50 percent of post-thyroidectomy patients with TCLM did not undergo RAI therapy, possibly due to the failure to meet therapeutic criteria, specifically, the lack of iodine uptake in metastatic lung lesions. In addition, the guidelines for RAI therapy of DTC [30] have indicated that ^131^I treatment for pulmonary metastasis is largely dependent of iodine uptake, age, nodule size, and the presence of other distant metastatic lesions. Notably, Song et al. have suggested that DTC patients at <40 years old and with small and only lung metastases, without other organ metastases, had better outcomes with RAI therapy [23]. Similarly, our study has identified that age plays a significant role in the effectiveness of RAI therapy on the prognosis of patients with lung metastasis after thyroidectomy. Specifically, our results have demonstrated that patients under 55 years of age treated with RAI therapy have a more favorable prognosis compared to those aged 55 years and above. Moreover, the study has revealed that patients with lung metastasis alone had a better survival rate than those with lung and other distant metastases after RAI therapy. Conversely, there was no statistically significant difference in prognosis among patients with multiple distant metastases, which may be attributed to the small sample size of patients with lung and other metastases. 

Additionally, it has been reported that some patients with thyroid carcinoma and lung metastases who either fail to uptake iodine or lose iodine uptake properties following RAI therapy eventually develop radioiodine-refractory thyroid cancer (RAIR-TC) over time [31]. The loss of iodine uptake may be consequent upon the dedifferentiation of malignant cells resulting in reduced expression of the sodium/iodide symporter (NIS) or clonal selection. One of the risk factors for the prognosis of these patients is the iodine uptake of distant metastases, Hong-Jun Song et al. found that, amongst TCLM patients, those unresponsive to RAI therapy bore a significantly lower 10-year survival rate [23]. However, only two-thirds of TCLM patients had ^131^I uptake in their metastases [32]. Considering the adverse effects of other therapeutic modalities, it is necessary to strictly grasp the indications for application before deciding on further treatment options. Thus, this analysis of various risk factors may assist in identifying patients who are suitable candidates for RAI treatment.

Finally, our findings support the 2015 ATA recommendations for RAI therapy [21] as a post-thyroidectomy treatment. However, it is worth noting that, while the 2015 ATA guidelines [21] strongly recommend RAI treatment for TCLM patients with lung micrometastases, our study has provided further evidence in specific subgroups of TCLM patients and has expanded the clinical application of RAI therapy for TCLM patients following primary site surgery.

Li et al. have reported that RAI therapy should be precisely administered to young patients with well-differentiated tumors and may improve survival rates of lung metastases [11]. Consistent with these findings, our study has demonstrated that RAI therapy significantly enhances OS and TCSS in TCLM patients following primary site surgery under the age of 55, PTC patients, and those with single organ metastases at lung. Additionally, the results may assist clinicians in determining which patients will benefit from RAI therapy, thus providing new avenues for treatment.

However, this study is not without certain limitations. First of all, the number of patients with ATC and MTC in the histology sample is limited, leading to potential statistical bias. Secondly, this study relies on a public database, and further validation of clinical information for patients is required. Additionally, the dose and frequency of administration, as well as the route of administration, the time interval between primary surgery and RAI treatment, and other factors may also influence RAI treatment outcomes. Unfortunately, the SEER database does not contain sufficient data to thoroughly investigate these issues. Finally, it should be noted that the SEER database does not include information on iodine uptake rate, thyroid-stimulating hormone suppression therapy, or serological indicators of thyroid, including serum *BRAFV600E* mutation, progressive nodal burden, extrathyroidal extension, thoracic surgery for lung metastasis, and thyroxine kinase inhibitors (TKI). Thoracic surgery for lung metastases can indeed potentially affect the efficacy of RAI treatment and may provide survival benefits for specific patients. Furthermore, postoperative survival rates depend highly on factors such as the primary tumor entity, histology and differentiation, absence of disease interval, and the number and size of metastatic lesions [33]. Research has demonstrated that targeted therapy with TKIs can improve clinical outcomes in TCLM patients [34,35]. These factors are essential for assessing the risk of thyroid cancer recurrence [36,37,38]. 

## 5. Conclusions

This study identified that, compared to primary site surgery alone, RAI in conjunction with surgery significantly improved TCSS for TCLM patients. Specifically, TCLM patients after thyroid surgery who are younger (<55 years), have PTC and metastases only in the lungs, and without involvement of other organs may benefit from RAI treatment. These results have important implications for identifying TCLM patients who have undergone primary site surgery that may be candidates for iodine therapy, as well as developing appropriate treatment regimens for them. 

## Figures and Tables

**Table 1:** Demographic and clinicopathologic features of thyroid cancer patients with lung metastases.

	All		Radioiodine Therapy				*p*
			Absence		Presence		
	(*n* = 609)		(*n* = 386)		(*n* = 223)		
Sex							
Female (*n*,%)	317	52.1%	201	52.1%	116	52.0%	<0.001
Male (*n*,%)	292	47.9%	185	47.9%	107	48.0%	
Race							
White (*n*,%)	463	76.0%	288	774.6%	175	78.5%	0.443
Black (*n*,%)	42	6.90%	30	7.80%	12	5.40%	
Other (*n*,%)	104	17.1%	68	17.6%	36	16.1%	
Marital status							
Single (*n*,%)	133	21.8%	74	19.2%	59	26.5%	0.010
Married (*n*,%)	343	56.3%	214	55.4%	129	57.8%	
Divorced (*n*,%)	44	7.20%	29	7.50%	15	6.70%	
Widowed (*n*,%)	89	14.6%	69	17.9%	20	9.00%	
Age (years)							
<55 (*n*,%)	177	29.1%	87	22.5%	90	40.4%	<0.001
≥55 (*n*,%)	432	70.9%	299	77.5%	133	59.6%	
Histology							
PTC (*n*,%)	369	60.6%	176	45.6%	193	193%	<0.001
MTC (*n*,%)	25	4.10%	25	6.50%	0	0.00%	
FTC (*n*,%)	67	11.0%	38	9.80%	29	13.0%	
ATC (*n*,%)	148	24.3%	147	38.1%	1	0.40%	
Tumor size (mm)							
≤10 (*n*,%)	29	4.80%	15	3.90%	14	6.30%	<0.001
11–20 (*n*,%)	69	11.3%	27	7.00%	42	18.8%	
21–40 (*n*,%)	160	26.3%	82	21.2%	78	35.0%	
>40 (*n*,%)	351	57.6%	262	67.9%	89	39.9%	
Stage							<0.001
II (*n*,%)	79	13.0%	25	6.50%	54	24.4%	
IV (*n*,%)	529	86.9%	360	93.3%	169	75.8%	
Unknown (*n*,%)	1	0.20%	1	0.30%	0	0.00%	
T							
T1 (*n*,%)	41	6.70%	16	4.10%	25	11.2%	<0.001
T2 (*n*,%)	44	7.20%	24	6.20%	20	9.00%	
T3 (*n*,%)	160	26.3%	59	15.3%	101	45.3%	
T4 (*n*,%)	352	57.8%	279	72.3%	73	32.7%	
TX (*n*,%)	9	1.50%	6	1.60%	3	1.30%	
T0 (*n*,%)	3	0.50%	2	0.50%	1	0.40%	
N							
N0 (*n*,%)	163	26.8%	111	28.8%	52	23.3%	<0.001
N1 (*n*,%)	416	68.3%	253	65.5%	163	73.1%	
NX (*n*,%)	30	4.90%	22	5.70%	8	3.60%	
Surgery of primary site							
No (*n*,%)	175	28.7%	174	45.1%	1	0.40%	<0.001
Yes (*n*,%)	434	71.3%	212	54.9%	222	99.6%	
LM							
Only lung (*n*,%)	440	72.2%	250	64.8%	190	85.2%	<0.001
Lung + 1(*n*,%)	129	21.2%	99	25.6%	30	13.5%	
Lung + 2 (*n*,%)	40	6.60%	37	9.60%	3	1.30%	

TNM categories, stage groups, and definitions used by SEER are based on the AJCC 7th Edition; LM: lung metastasis; Only lung: TC patients with single organ metastases at lung; Lung + 1: TC patients combined with lung and other one distant metastasis; Lung + 2: TC patients combined with lung and other several distant metastases.

**Table 2:** Univariate and multivariable Cox analysis of overall survival and thyroid cancer-specific survival in TCLM patients.

Variable	Univariate Analysis				Multivariate Analysis			
	Overall Survival		Specific Survival		Overall Survival		Specific Survival	
	HR (95%CI)	*p*	HR (95%CI)	*p*	HR (95%CI)	*p*	HR (95%CI)	*p*
Sex								
Female	Ref		Ref		/		/	
Male	1.027 (0.835–1.263)	0.802	0.980 (0.789–1.217)	0.855	/	/	/	/
Race								
White	Ref		Ref		/		/	
Black	0.668 (0.419–1.063)	0.088	0.737 (0.463–1.175)	0.200	/	/	/	/
Other	0.908 (0.683–1.207)	0.506	0.908 (0.673–1.223)	0.524	/	/	/	/
Marital status								
Single	Ref		Ref		/		/	
Married	0.406 (0.287–0.576)	<0.001	1.593 (1.173–2.164)	0.003	/	/	/	/
Divorced	0.639 (0.485–0.843)	0.002	1.559 (0.969–2.509)	0.067	/	/	/	/
Widowed	0.633 (0.407–0.985)	0.043	2.660 (1.848–3.827)	<0.001	/	/	/	/
Age (years)								
<55	Ref		Ref		Ref		Ref	
≥55	2.474 (1.885–3.246)	<0.001	2.264 (1.715–2.988)	<0.001	1.343 (0.989–1.823)	0.059	1.225 (0.897–1.672)	0.201
Histology								
PTC	Ref		Ref		Ref		Ref	
MTC	2.233 (1.350–3.695)	0.002	2.333 (1.386–3.925)	0.001	0.892 (0.519–1.534)	0.680	0.917 (0.523–1.605)	0.761
FTC	1.578 (1.118–2.229)	0.010	1.425 (0.974–2.085)	0.068	1.231 (0.848–1.788)	0.275	1.124 (0.747–1.691)	0.575
ATC	10.827 (8.256–14.200)	<0.001	11.005 (8.316–14.564)	<0.001	3.981 (2.876–5.512)	<0.001	3.864 (2.763–5.403)	<0.001
Tumor size (mm)								
≤10	Ref		Ref		Ref		Ref	
11–20	1.265 (0.634–2.526)	0.505	1.044 (0.514–2.123)	0.905	1.462 (0.704–3.038)	0.308	1.222 (0.577–2.588)	0.601
21–40	1.381 (0.732–2.607)	0.319	1.193 (0.628–2.266)	0.590	1.117 (0.549–2.271)	0.761	1.023 (0.499–2.097)	0.952
>40	2.868 (1.566–5.252)	0.001	2.612 (1.425–4.786)	0.002	1.397 (0.708–2.756)	0.335	1.228 (0.618–2.437)	0.558
Stage								
II	Ref		Ref		Ref		Ref	
IV	7.143 (3.915–13.036)	<0.001	6.996 (3.724–13.144)	<0.001	2.794 (1.433–5.447)	0.003	2.768 (1.379–5.556)	0.004
Unknown	17.813 (2.210–133.618)	0.007	17.65 (2.251–138.415)	<0.001	1.698 (0.161–17.934)	0.660	2.087 (0.182–23.883)	0.554
T								
T1	Ref		Ref		Ref		Ref	
T2	1.212 (0.576–2.548)	0.612	0.947 (0.402–2.229)	0.900	1.068 (0.448–2.548)	0.881	0.791 (0.297–2.105)	0.639
T3	1.043 (0.569–1.914)	0.892	1.044 (0.540–2.018)	0.899	1.028 (0.517–2.043)	0.938	1.012 (0.482–2.127)	0.975
T4	4.238 (2.425–7.405)	<0.001	4.614 (2.519–8.451)	<0.001	1.798 (0.922–3.507)	0.085	1.868 (0.910–3.832)	0.088
TX	2.111 (0.752–5.926)	0.156	1.965 (0.625–6.177)	0.248	0.895 (0.272–2.947)	0.855	0.731 (0.190–2.817)	0.649
T0	0.874 (0.114–6.692)	0.897	1.076 (0.139–8.333)	0.944	0.663 (0.080–5.496)	0.703	0.734 (0.088–6.140)	0.775
N								
N0	Ref		Ref		Ref		Ref	
N1	1.061 (0.836–1.346)	0.627	1.083 (0.843–1.391)	0.533	1.260 (0.972–1.634)	0.081	1.225 (0.933–1.608)	0.144
NX	1.931 (1.224–3.048)	0.005	2.041 (1.276–3.264)	0.003	1.771 (1.084–2.895)	0.023	1.848 (1.114–3.066)	0.017
LM								
Only lung	Ref		Ref		Ref		Ref	
Lung + 1	1.635 (1.282–2.084)	<0.001	1.679 (1.304–2.162)	<0.001	1.299 (1.002–1.684)	0.048	1.359 (1.039–1.779)	0.025
Lung + 2	2.505 (1.741–3.604)	<0.001	2.622 (1.809–3.800)	<0.001	1.347 (0.917–1.978)	0.129	1.403 (0.948–2.076)	0.091
Surgery of primary site								
No	Ref		Ref		Ref		Ref	
Yes	0.223 (0.179–0.278)	<0.001	0.224 (0.178–0.282)	<0.001	0.653 (0.506–0.842)	0.001	0.664 (0.510–0.864)	0.002
RAI								
No	Ref		Ref		Ref		Ref	
Yes	0.188 (0.142–0.248)	<0.001	0.169 (0.124–0.229)	<0.001	0.397 (0.283–0.558)	<0.001	0.371 (0.257–0.535)	<0.001

TNM categories, stage groups, and definitions used by SEER are based on the AJCC 7th Edition. LM: lung metastasis; Only lung: TC patients with single organ metastases at lung; Lung + 1: TC patients combined with lung and other one distant metastasis; Lung + 2: TC patients combined with lung and other several distant metastases; RAI: radioiodine therapy.

**Table 3:** Multivariable Cox analysis of overall survival and thyroid cancer-specific survival in TCLM patients following surgery of primary site.

Variable	N (%)	Overall Survival		Specific Survival	
		HR (95%CI)	*p*	HR (95%CI)	*p*
Age (years)					
<55	156 (35.9%)	Ref		Ref	
≥55	278 (64.1%)	1.484 (1.018–2.165)	0.040	1.379 (0.937–2.028)	0.103
Histology					
PTC	316 (72.8%)	Ref		Ref	
MTC	13 (3.0%)	1.038 (0.485–2.220)	0.924	1.087 (0.505–2.342)	0.831
FTC	50 (11.5%)	1.396 (0.890–2.189)	0.146	1.284 (0.779–2.116)	0.328
ATC	55 (12.7%)	4.672 (2.964–7.366)	<0.001	4.626 (2.890–7.405)	<0.001
Tumor size (mm)					
≤10	25 (5.8%)	Ref		Ref	
11–20	61 (14.1%)	0.964 (0.421–2.211)	0.932	0.839 (0.358–1.962)	0.685
21–40	129 (29.7%)	0.781 (0.344–1.777)	0.556	0.673 (0.292–1.552)	0.354
>40	219 (50.5%)	0.952 (0.432–2.097)	0.903	0.808 (0.363–1.799)	0.602
Stage					
II	77 (17.7%)	Ref		Ref	
IV	357 (82.3%)	2.631 (1.238–5.592)	0.012	2.589 (1.170–5.728)	0.019
T					
T1	36 (8.3%)	Ref		Ref	
T2	32 (7.4%)	1.213 (0.385–3.818)	0.742	0.836 (0.216–3.231)	0.795
T3	147 (33.9%)	1.516 (0.648–3.545)	0.337	1.501 (0.604–3.731)	0.382
T4	213 (49.1%)	3.133 (1.366–7.187)	0.007	3.358 (1.386–8.133)	0.007
TX	4 (0.9%)	2.892 (0.588–14.221)	0.191	1.757 (0.208–14.854)	0.605
T0	2 (0.5%)	0.000 (–)	0.951	0.000 (–)	0.955
LM					
Only lung	329 (75.8%)	Ref		Ref	
Lung + 1	85 (19.6%)	1.551 (1.097–2.194)	0.013	1.725 (1.207–2.464)	0.003
Lung + 2	20 (4.6%)	1.866 (1.064–3.274)	0.03	1.837 (1.026–3.288)	0.041
RAI					
No	212 (48.8%)	Ref		Ref	
Yes	222 (51.2%)	0.507 (0.350–0.733)	<0.001	0.482 (0.323–0.718)	<0.001

TNM categories, stage groups, and definitions used by SEER are based on the AJCC 7th Edition. LM: lung metastasis; Only lung: Thyroid cancer patients with single organ metastases at lung; Lung + 1: Thyroid cancer patients combined with lung and other one distant metastasis; Lung + 2: Thyroid cancer patients combined with lung and other several distant metastases; RAI: radioiodine therapy.

## Data Availability

These researchers analyzed publicly available datasets. This information can be found here: “https://seer.cancer.gov/”.

## Supplementary Materials

The following supporting information can be downloaded at: https://www.mdpi.com/article/10.3390/biomedicines12030532/s1, Figure S1: Stratified Kaplan-Meier survival curves of TCSS based on subgroup of age and histology; Table S1: Demographic and clinicopathologic features of thyroid cancer patients with and without lung metastases; Table S2: Surgical types of primary site in TCLM patients.

## Abbreviations

Thyroid cancer (TC); Differentiated thyroid cancer patients with lung metastasis (DTC–LM); Journal of the National Cancer Center (JNCC); differentiated thyroid cancer (DTC); medullary thyroid cancer (MTC); anaplastic thyroid cancer (ATC); Papillary thyroid cancer (PTC); follicular thyroid cancer (FTC); Radioiodine therapy (RAI); thyroid cancer with lung metastasis (TCLM); American Thyroid Association (ATA); The Surveillance, Epidemiology, and End Results (SEER); American Joint Committee on Cancer (AJCC); Collaborative Staging (CS); odds ratio (OR); overall survival (OS); thyroid cancer-specific survival (TCSS); hazard ratio (HR); lung metastasis (LM); confidence intervals (CI); radioiodine-refractory thyroid cancer (RAIR-TC); thyroxine kinase inhibitors (TKI).
