# Supplementary material for: The Effect of Radioiodine Therapy on the Prognosis of Differentiated Thyroid Cancer with Lung Metastases
Source: Biomedicines. 2024 Feb 27;12(3):532. doi: 10.3390/biomedicines12030532 (PMC10967879; doi:10.3390/biomedicines12030532)
Supplement: Supplementary file 1 [file biomedicines-12-00532-s001.zip › Supplement information.pdf]

a

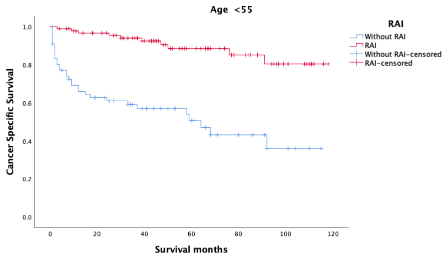


b

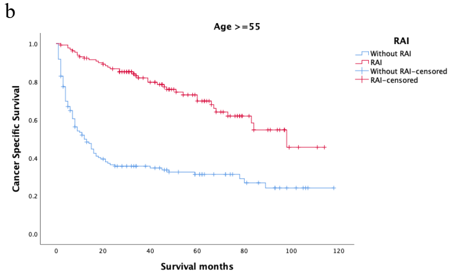


| Age | RAI | Mean | | | p |
|---|---|---|---|---|---|
| | | Estimate | 95% Confidence Interval | | |
| | | | Lower Bound | Upper Bound | |
| <55 | Without RAI | 60.380 | 47.406 | 73.354 | <0.001 |
| | RAI | 105.202 | 97.857 | 112.547 | |
| >=55 | Without RAI | 41.263 | 32.891 | 49.635 | <0.001 |
| | RAI | 81.520 | 73.354 | 89.687 | |

c

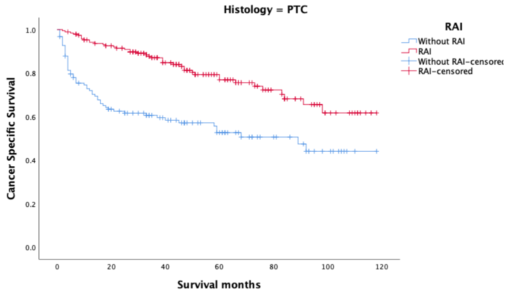


d

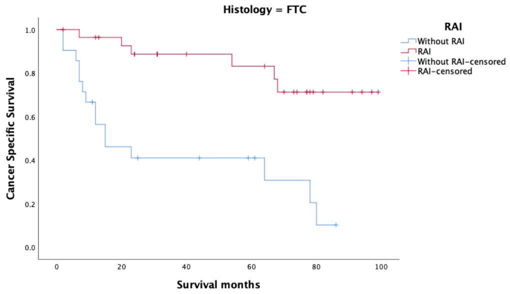


| Histology | RAI | Mean | | | p |
|---|---|---|---|---|---|
| | | Estimate | 95% Confidence Interval | | |
| | | | Lower Bound | Upper Bound | |
| PTC | Without RAI | 66.702 | 56.997 | 76.408 | <0.001 |
| | RAI | 92.966 | 86.434 | 99.498 | |
| FTC | Without RAI | 37.491 | 22.617 | 52.364 | <0.001 |
| | RAI | 83.498 | 72.398 | 94.598 | |

**Figure S1. Stratified Kaplan-Meier survival curves of TCSS based on subgroup of age and histology**
