# Supplementary material for: The Effect of Radioiodine Therapy on the Prognosis of Differentiated Thyroid Cancer with Lung Metastases
Source: Biomedicines. 2024 Feb 27;12(3):532. doi: 10.3390/biomedicines12030532 (PMC10967879; doi:10.3390/biomedicines12030532)
Supplement: Supplementary file 1 [file biomedicines-12-00532-s001.zip › Supplement Table.pdf]

Table S1. Demographic and clinicopathologic features of thyroid cancer patients with and without lung metastases.

	All		Lung metastasis				*p*
			Absence		Presence		
	(n=68709)		(n=68100)		(n=609)		
Sex							
Female (*n*,%)	53072	77.2%	52755	77.5%	317	52.1%	<0.001
Male (*n*,%)	15637	22.8%	15345	22.5%	292	47.9%	
Race							
White (*n*,%)	54775	79.7%	54312	79.8%	463	76.0%	0.053
Black (*n*,%)	4414	6.4%	4372	6.4%	42	6.9%	
Other (*n*,%)	9520	13.9%	9416	13.8%	104	17.1%	
Marital status							
Single (*n*,%)	16885	24.6%	16752	24.6%	133	21.8%	<0.001
Married (*n*,%)	44108	64.2%	43765	64.3%	343	56.3%	
Divorced(*n*,%)	4920	7.2%	4876	7.2%	44	7.2%	
Widowed (*n*,%)	2796	4.1%	2707	4.0%	89	14.6%	
Age(years)							
<55 (*n*,%)	45523	66.3%	45346	66.6%	177	29.1%	<0.001
≥55 (*n*,%)	23186	33.7%	22754	33.4%	432	70.9%	
Histology							
PTC (*n*,%)	63946	93.1%	63577	93.4%	369	60.6%	
MTC (*n*,%)	1068	1.6%	1043	1.5%	25	4.1%	<0.001
FTC (*n*,%)	3293	4.8%	3226	4.7%	67	11.0%	
ATC (*n*,%)	402	0.6%	254	0.4%	148	24.3%	
Tumor size(mm)							
≤10 (*n*,%)	25800	37.5%	25771	37.8%	29	4.8%	
11-20 (*n*,%)	19803	28.8%	19734	29.0%	69	11.3%	<0.001
21-40 (*n*,%)	15718	22.9%	15558	22.8%	160	26.3%	
>40 (*n*,%)	7388	10.8%	7037	10.3%	351	57.6%	
Stage							<0.001
I (*n*,%)	48837	71.1%	48837	71.7%	0	0.0%	
II (*n*,%)	4594	6.7%	4515	6.6%	79	13.0%	
III (*n*,%)	8542	12.4%	8542	12.5%	0	0.0%	<0.001
IV (*n*,%)	4662	6.8%	4133	6.1%	529	86.9%	
Unknown (*n*,%)	2074	3.0%	2073	3.0%	1	0.2%	
T							<0.001
T1 (*n*,%)	40106	58.4%	40065	58.8%	41	6.7%	
T2 (*n*,%)	11647	17.0%	11603	17.0%	44	7.2%	
T3 (*n*,%)	14228	20.7%	14068	20.7%	160	26.3%	<0.001
T4 (*n*,%)	2328	3.4%	1976	2.9%	352	57.8%	
TX (*n*,%)	307	0.4%	298	0.4%	9	1.5%	

T0 (*n*,%)	93	0.1%	90	0.1%	3	0.5%	
N							
N0 (*n*,%)	48794	71.0%	48631	71.4%	163	26.8%	
N1 (*n*,%)	17382	25.3%	16966	24.9%	416	68.3%	<0.001
NX (*n*,%)	2533	3.7%	2503	3.7%	30	4.9%	
Surgery of primary site							
No (*n*,%)	1158	1.7%	983	1.4%	175	28.7%	<0.001
Yes (*n*,%)	67551	98.3%	67117	98.6%	434	71.3%	
RAI							
No (*n*,%)	39594	57.6%	39208	57.6%	386	63.4%	0.004
Yes (*n*,%)	29115	42.4%	28892	42.4%	223	36.6%	
EBRT							
No (*n*,%)	67692	98.5%	67282	98.8%	410	67.3%	<0.001
Yes (*n*,%)	1017	1.5%	818	1.2%	199	32.7%	
Chemotherapy							
No (*n*,%)	68246	99.3%	67782	99.5%	464	76.2%	<0.001
Yes (*n*,%)	463	0.7%	318	0.5%	145	23.8%	

RAI: radioiodine therapy; EBRT: External beam radiotherapy; TNM categories, stage groups, and definitions used by SEER are based on the AJCC 7th Edition.

Table S2. Surgical types of primary site in TCLM patients.

Surgical types of primary site	Radioiodine therapy		ALL
	Absence	Presence	
Total thyroidectomy/Subtotal or near total thyroidectomy	169	211	380
	79.7%	95.0%	87.6%
Local surgical excision	38	10	48
	17.9%	4.5%	11.1%
Unknown	5	1	6
	2.4%	0.5%	1.4%
